# Relocation preference and settlement: Lessons from the Poverty Alleviation Relocation Program in China

**DOI:** 10.1371/journal.pone.0309534

**Published:** 2024-10-04

**Authors:** Shuai Zhou, Guangqing Chi, Huanguang Qiu, Zhen Lei, Erica A. H. Smithwick, Jiquan Chen

**Affiliations:** 1 Department of Agricultural Economics, Sociology, and Education, The Pennsylvania State University, University Park, Pennsylvania, United States of America; 2 Department of Global Development, Cornell University, Ithaca, New York, United States of America; 3 School of Agricultural Economics and Rural Development, Renmin University of China, Beijing, China; 4 Department of Energy and Mineral Engineering, The Pennsylvania State University, University Park, Pennsylvania, United States of America; 5 Department of Geography and the Earth and Environmental Systems Institute, The Pennsylvania State University, University Park, Pennsylvania, United States of America; 6 Department of Geography, Environment, and Spatial Sciences, Michigan State University, East Lansing, Michigan, United States of America; Sichuan Agricultural University, CHINA

## Abstract

We explored the linkages between socioeconomic and demographic factors, relocation preference, and settlement associated with China’s Poverty Alleviation Relocation Program. Using multivariate ordinal logistic regression, panel data modeling, and multilevel methods, we found that outdated infrastructure at places of origin, such as long distances to paved roads or elementary schools, increased the probability of relocation, and the presence of left-behind household members at the origin compromised re-settlement. This study sheds light on the community- and household-level factors that influence relocation preference and settlement, offering valuable insights for future research and informing the design and implementation of relocation projects.

## Introduction

Relocation refers to a form of mobility characterized by the geographic displacement of individuals from one location to another. The term is often used interchangeably with migration, displacement, evacuation, and planned relocation [1, 2]. In recent decades, relocation has been employed as a strategy to facilitate infrastructure development [3, 4] and address challenges such as resource scarcity [5], poverty [6, 7], access to education and health care [8, 9], and climate-induced disasters [10, 11]. Development and problem solving—oriented relocations involving large populations are usually led by governments. One notable example of this is the relocation associated with the Three Gorges Dam—a government-led project in China designed to control floods and transform the environment—which resulted in the displacement of approximately six million people [12, 13].

Similar to migration, relocation can manifest in various forms. Depending on voluntariness and whether the movements occur across borders, relocation can be categorized as self-driven, forced, internal, and international relocation. Previous studies on relocation have primarily focused on self-driven relocation, particularly employment-related moves [14, 15]. They have identified various factors that influence willingness to relocate, including individual characteristics such as gender and marital status [16], place-level factors such as the similarity between places of origin and destination [17, 18], and the availability of financial incentives [19]. It is worthwhile to distinguish self-driven relocation and internal migration, two common geographical movements in human societies. Although both are forms of human mobility and share common drivers such as economic opportunities, improved living conditions, access to services, and social networks, they exhibit notable differences. Self-driven relocation involves individual agency and choices in the decision-making process, placing a strong emphasis on the active role of individuals in shaping their moves. In contrast, internal migration is triggered by a broader range of motivations, including economic, social, and environmental factors. Additionally, self-driven relocation is typically a voluntary action taken by individuals, reflecting their personal preferences and aspirations, while internal migration can encompass both voluntary and involuntary or forced movements that are influenced by factors such as conflict, environmental disasters, or government policies.

Other research has focused on government-led relocation projects, primarily aiming to evaluate the efficacy of such relocations and to assess their impacts on the involved populations. For example, HOPE VI and Moving to Opportunity are two well-known federal-led relocation projects in the United States. Studies of these two projects have generally suggested that relocation has had limited impacts on the participants’ financial, educational, and social well-being [6, 20, 21]. In recent decades, relocation has been increasingly driven by environmental disasters such as floods [22] and hurricanes [23, 24]. However, disaster-related relocation research often focuses on relocation and settlement separately. To the best of our knowledge, studies exploring the driving forces in relocation preference following government-led subsidized relocation projects, as well as settlement—the state of remaining residentially stable at destination communities—in one research design from socioeconomic and demographic perspectives are limited. The requisite data for a comprehensive analysis of relocation preference and settlement, including pre- and post-move records, are rarely available.

We attempt to fill this knowledge gap by systemically investigating the socioeconomic and demographic characteristics associated with relocation preference and settlement within the Poverty Alleviation Relocation Program (PARP), a part of China’s Targeted Poverty Alleviation Project (TPAP). The investigation intso the underlying determinants that shape relocation preferences and settlement patterns within the PARP offers a valuable opportunity to gain an understanding of the factors influencing individuals’ decisions to engage in government-led relocation projects and their subsequent settlement in the new communities. This research is of particular relevance in the context of global environmental change and escalating regional conflicts (e.g., the Ukrainian crisis), which are expected to cause a substantial increase in the frequency of relocations. Moreover, this research provides policymakers with critical insights for the effective formulation and implementation of relocation policies, thereby promoting the overall well-being of individuals involved in the PARP and offering guidance for similar relocation programs in the future.

First announced in 2014 and officially initiated in 2016, the TPAP aimed to lift about 70 million people out of extreme poverty by 2020. This involved the relocation of approximately ten million people from 1,400 targeted counties in 22 provinces that were characterized by high poverty rates and environmental vulnerability according to the PARP, the largest relocation project in recent history anywhere in the world in terms of the population involved [25, 26]. The first step of the PARP was the identification of impoverished populations. The government used an annual net individual income of 2,300 RMB (approximately 362 USD at the 2014 exchange rate) as the poverty threshold [25]. Populations earning below this threshold were registered, and their information was archived in the National Poverty Alleviation Information System for monitoring their poverty status based on the pre-defined threshold. One approach to relocation aimed to preserve the original social networks and support systems of the relocatees to enhance their participation. This involved resettling all the poor households from a village in a single location. However, it was not always feasible to accommodate an entire targeted population in one settlement, so some cohorts were dispersed across multiple settlements. During the relocation process, the government provided subsidies to targeted populations as incentives, usually in the form of government-built apartments (without property rights) proportional to household size and/or in-kind benefits in the relocation destination. Relocations usually happened within the same jurisdiction to reduce moving costs and social integration issues. Government officials visited targeted populations to disseminate detailed information about the PARP’s policies, aiming to increase their understanding and encourage participation. After relocation, participants were provided with community service work opportunities and training to bolster their employment prospects in the new community. Ultimately, households had the right to decide whether or not to relocate. In sum, PARP was a government-led and subsidized voluntary relocation program that enables us to examine the driving forces of the targeted population’s relocation preferences and settlement at the destination.

In the literature on relocation, prior studies have shown that factors such as community similarities, financial incentives, and demographic characteristics including age, gender, and income significantly influence relocation willingness. For example, Noe and Barber [17] found that people were more likely to relocate to communities that were similar to their original locations. Wagner and Westaby [18] found similar results, with cultural similarities between pre- and post-relocation communities being associated with higher levels of relocation willingness. They also found that financial incentives were the most important factor in explaining the differences in relocation willingness. Abraham, Bähr, and Trappmann [16] found that gender and marital status affected relocation willingness: married women had a lower willingness to relocate than married men; however, there was no difference in relocation willingness between single women and single men. Konopaske, Robie, and Ivancevich [27] also found that spouses’/significant others’ willingness to relocate played an important role in deciding whether or not to relocate. Lo and Wang [28] investigated the level of voluntary participation among TPAP participants and found a strong willingness for relocation, particularly among younger individuals, those with higher wealth, and those with off-farm employment opportunities.

In recent decades, environmental disasters such as hurricanes and floods have increasingly influenced relocation or evacuation. Studies have identified a bundle of socioeconomic and demographic factors that influence disaster-affected populations’ relocation or evacuation practices. For example, Bukvic and Owen [23] surveyed 46 households from highly affected coastal communities five months after Hurricane Sandy and explored their attitudes toward relocation. Their results suggested that respondents were aware of climate-related coastal risks such as hurricanes and sea-level rise and were willing to relocate to other places, mainly for health and safety reasons. In a relocation study from disaster-threatened areas in rural China, Xu et al. [29] found that sense of place significantly decreased relocation willingness, while risk perception significantly increased the probability of relocation. Not all disaster-threatened populations are relocatable. For instance, Mavhura et al. [22] conducted interviews with residents in a flood-prone community in Zimbabwe and found that, although relocation was considered a viable strategy to cope with floods, the lack of compensation for their lost land and livelihoods, along with the inadequate basic infrastructure in relocation sites such as schools and clinics, posed significant barriers to the relocation of the flood-affected population. Thiede and Brown [24] examined relocation behavior prior to Hurricane Katrina and found that Black and less-educated populations were least likely to relocate due to a lack of necessary resources such as transportation and financial means to initiate relocation.

Like migration, relocations are often coupled with counterstream return flows that undermine settlement. As mentioned earlier, settlement generally refers to the state of remaining residentially stable at a relocation destination. Previous studies have found two main mechanisms through which settlement can be undermined in government-led relocation projects. First, insufficient support at the relocation destination can destabilize government-led relocation. In an early study of the Employment Transfer Scheme in Scotland, a government-led labor-mobility project, Beaumont [30] found that 24.8% of the sampled population returned. The primary reasons for returns were insecure financial situations and the unavailability of satisfactory housing in the destination community. Second, the failure to achieve relocation goals may affect a settlement. In a comparative analysis of the HOPE VI and the Moving to Opportunity programs, Goetz [6] found limited evidence to support the notion that relocation positively impacted participants’ living conditions, neighborhood satisfaction, and employment security. It was also suggested that these limited improvements can potentially have an indirect destabilizing effect on the overall relocation process. Regarding disaster relocation, Groen and Polivka [31] found that returns vary across demographic groups, with Blacks, young adults, and single individuals being less likely to return to their place of origin after Hurricane Katrina, likely because their residences and neighborhoods experienced greater physical damage than those of other demographic groups.

## Conceptual framework

In essence, relocation is a geographic movement of populations, making it analogous to migration. Previous studies have theoretically framed and empirically investigated relocation from a migration perspective [1, 2]. We followed this research convention and conceptualized relocation as a migratory response to unfavorable living and environmental conditions. Classical migration theory attributes initial, repeat, and return migrations to different driving factors at places of origin and destinations and a series of obstacles such as distance between the two ends [32, 33]. In the twentieth century, the theoretical investigation of migration from economic perspectives gained popularity, with neoclassical economics (NE) and the new economics of labor migration (NELM) as the two main theoretical frameworks. NE theory suggests that the markets at both the sending and the destination community are well-functioning but in different stages of development; migrants flow to geographically advantaged and well-developed labor markets for higher wages to maximize their incomes and well-being [34]. NE attributes return migration to financial failure at the destination. In summary, NE considers migration to be an income- or utility-maximizing behavior [35]. In response to such narrow foci, NELM contends that migration is driven by the interest of not only increasing income but also minimizing risk through diversifying sources of income [36, 37]. Massey and Espinosa [34] espoused that migration occurs not only to seek higher earnings but also to avoid market failure at places of origin and to accumulate human capital at places of destination. In a comprehensive examination of theoretical perspectives on migration and development, De Haas [38] also emphasized the significance of social and migrant networks in facilitating additional migration flows. Empirical studies have consistently demonstrated the influential role of these networks on migration dynamics both at the global level [39] and within specific regions, such as the migrations from Mexico to the United States [40].

While the aforementioned theoretical frameworks focused on either structural factors or the agency of actors, they neglected the interrelationship and feedback mechanisms between them [41–43]. The migration system theory was developed to incorporate structural factors and actors’ agencies within the migration process [44]. Specifically, the migration system theory takes a systematic approach and uses all the elements from the places of origin, destinations, and origin—destination combinations to study migration decision-making instead of separating places of origin and destination [45]. In doing so, the migration system theory bridges the micro—macro gap in the migration process by filling it up with the interactions and feedback mechanisms between individual actors and the broader environment [46]. For example, information about migrants’ situations at the destination can be transmitted to the place of origin, affecting further migrations, depending on whether the feedback is positive or negative [41].

**Fig 1:**
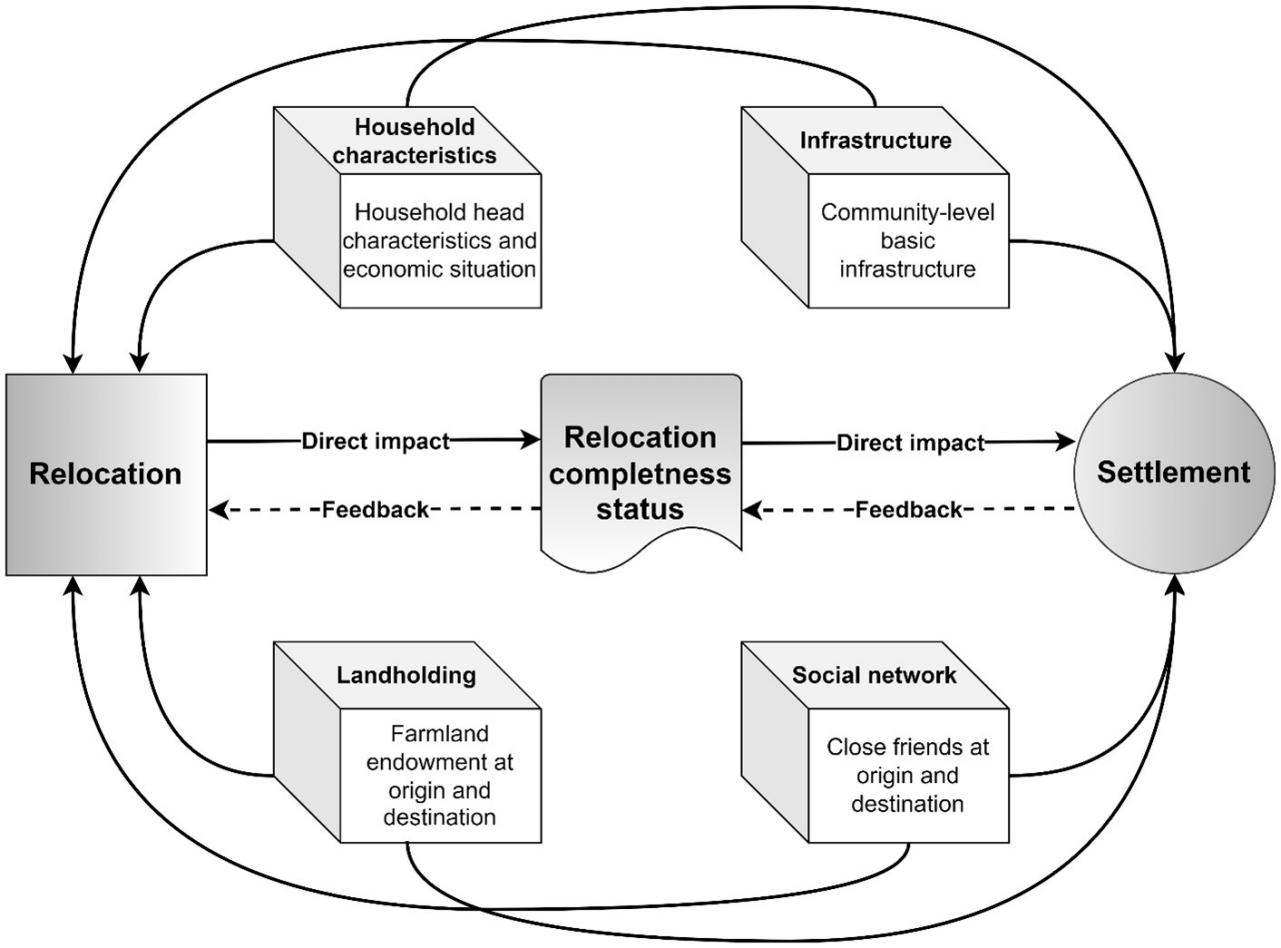
The conceptual framework for exploring relocation preference and settlement in the Poverty Alleviation Relocation Program (PARP) in China. Solid arrows show the direct impacts of covariates on relocation and settlement, respectively, while dashed arrows suggest a feedback mechanism that affects relocation and its subsequent settlement.

Building on De Haas’s [38] theoretical approach, we developed a conceptual framework (Fig 1) that delineates the relationships and feedback mechanisms among various factors within the relocation—settlement system. Household characteristics, landholding, infrastructure, and social networks at both origin and destination locations individually influenced the relocation and settlement decision-making of the targeted population in the PARP project. Simultaneously, the act of relocation itself impacted the cohesion of households at both places of origin and destination, subsequently influencing the settlement outcomes of the relocatees.

## Hypotheses

Drawing on theoretical and empirical evidence and our conceptual framework, we developed three specific hypotheses regarding the factors associated with relocation preference and settlement.

**Hypothesis 1: Basic infrastructure at the place of origin affects relocation preference. Specifically, the distance to transportation and educational infrastructure increases relocation preference.** The classic push—pull theory and the migration system approach emphasize the effect of community characteristics in deciding where to move [32, 41]. This implies that the basic infrastructure of the community is one of the major concerns that affect migrants’ relocation preference—people prefer places with higher transportation accessibility, more educational resources, and other easily accessible advantages. As such, we expected a positive association between relocation preference and the distance to basic infrastructure such as paved roads and schools.
**Hypothesis 2: Landholding size negatively affects relocation preference; the more the landholding size, the less likely the household will relocate to a new settlement.** In the Chinese context, landholding size has been found to be related to people’s movement [47]. Agriculture has a long-standing history in China, and it remains a crucial livelihood strategy for rural households. Research indicates that larger landholdings are associated with a reduced likelihood of rural households migrating to urban areas in China [48]. Despite the recent increase in rural—urban migration and the diversification of income sources through off-farm activities, challenges persist because of the existing population registration system, which prevents rural residents from obtaining urban citizenship and equal access to social welfare benefits [49, 50]. Consequently, the connection between rural residents and their farmland may remain strong because farmland and associated agricultural earnings provide a degree of financial stability. Therefore, we expected that landholding size would decrease relocation preference.
**Hypothesis 3: Relocation completeness plays an important role in determining settlement at the destinations. Compared with those who have relocated their entire household, households with left-behind members are more likely to visit their places of origin after the relocation.** As for the counterstream of migratory flow embedded in the migration system, return migration can be attributed to similar micro- and meso-level characteristics as the initial migration. However, in this specific relocation project, the importance of left-behind members is particularly emphasized in determining settlement. The PARP encourages the movement of the entire household. In reality, both internal and international migration sometimes start with young adults migrating first, then bringing children and the elderly, eventually finishing with a reunion of the household at the place of destination [51, 52]. Similar patterns were observed in the PARP: fieldwork observations during the survey indicated that young couples and their children were the first to move, while some elderly populations were temporarily left behind at their places of origin. The separation of household members and the interruption of family ties may lead to relocated households temporarily revisiting their places of origin. Consequently, we hypothesized that households with left-behind household members at their places of origin would experience greater instability following relocations.

## Materials and methods

### Data

**Fig 2:**
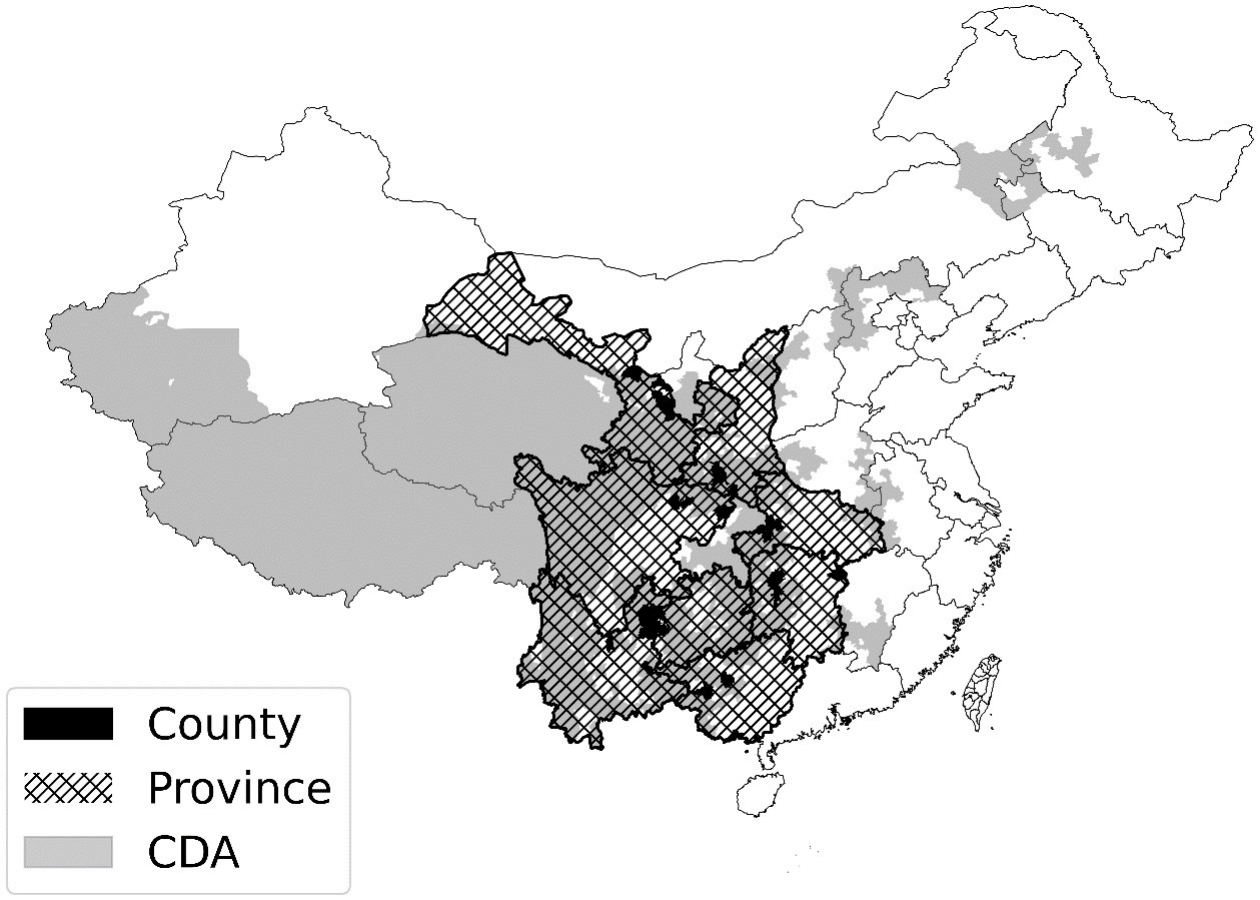
Distribution of the contiguous destitute areas (CDAs), including the eight sampled provinces and 16 sampled counties. The data for this figure comes from Natural Earth. This figure is for illustrative purposes only.

We conducted three waves of surveys (2016, 2017, and 2019). The 2016 survey examined the driving forces of relocation willingness because relocation had not yet commenced in that year. In the 2017 and 2019 surveys, given that relocation had started and some households had arrived at their destinations, our objective was to investigate the driving forces behind settlement after relocation. Using a multistage sampling strategy, we selected our baseline survey sample from the National Poverty Alleviation Information System in 2016, the official poverty dataset maintained by the central government of China. First, we purposely selected eight provinces (Gansu, Shaanxi, Sichuan, Yunnan, Guizhou, Guangxi, Hunan, and Hubei) from the contiguous destitute areas (CDAs) [53] where poverty is clustered. These selected provinces cover the most southwestern parts of the CDAs (see Fig 2). Second, we randomly selected two government-identified poor counties from each of the eight provinces. Finally, we randomly selected 144 households from the selected counties. This led to a total of 2,304 households being selected for participation. These 2,304 households were interviewed using a household survey (see S1 File for an excerpt of the survey instruments). We used an additional questionnaire to collect contextual information at the community level, which was usually filled out by village committee members most familiar with their communities. We hired trained college and graduate students to assist with the face-to-face data collection.

We collected 2,185 responses from the 2016 wave (95% of the selected households). In the second wave of the survey in 2017, we tracked down 1,898 households (about 87% of the households surveyed in 2016). Among those 1,898 households, only 475 households (25%) had relocated, likely because the relocation program was in its infancy in 2017. In the third wave of the survey in 2019, using both face-to-face and phone interviews, we tracked down 2,034 of the households surveyed in 2016, among which 1,147 had relocated (56%). We used the first wave of the study for the analysis of relocation preference and combined the second and third waves of the study to explore the driving forces of the relocatees’ settlement. Our questionnaire and survey procedures for data collection from the sampled households were approved by the ethics committee at Renmin University of China (Grant #71861147002). Prior to the interviews, informed oral consent was obtained from each participant. Trained interviewers ensured that all participants were fully informed about the study’s objectives and the protection of their privacy, confidentiality, and anonymity. To safeguard participant confidentiality, all personal information that could potentially identify individuals was anonymized to prevent any traceability.

After data cleaning, we got 2,146 valid samples for 2016 and 1,662 for 2017 and 2019 in combination. It is important to note that some missing data were identified in our samples, particularly pertaining to household head information and household income. Specifically, in the 2016 samples, eight households (0.37%) had missing data on household head information and five households (0.23%) had missing data on household income. In the 2019 samples, 14 households (1.22%) had missing data on household head information and four households (0.35%) had missing data on household income. Missing values for household heads usually happened when a household consisted of a widow and her children and no household head was reported. In those cases, we designated the widow as the household head and input household head information accordingly. If the widow was considered too old to make decisions for the household (age > 60), we designated the oldest child as the household head and filled out the missing information accordingly. For missing data on household income, we used unconditional mean imputation to fill in the missing information. For instance, if the household did not report its household income, the mean household income in the village to which the household belonged was used instead. The mean-based imputation of less than 1% of the samples did not significantly affect the results, especially when considering that these households are government-certified poor households from homogeneous areas who share many common traits, including financial situations. Another thing worth noting is that the research design did not incorporate weighting; consequently, we did not apply weight to the analysis.

### Variables

#### Dependent variables

Our dependent variables are relocation preference and settlement. We defined relocation preference as the likelihood of relocating to another place. In the survey we conducted in 2016, we asked the question, “How much would you like to relocate to the new settlement?” As the targeted populations might not have information about their exact relocation sites at this stage, the question aimed to assess their general willingness towards relocation, despite the lack of specific details about the relocation sites. Answers were coded from 1 to 5, where 1 represents “not likely” to relocate and 5 represents “very likely” to relocate. We used the answers to this question to denote relocation preferences.

In the relocation context, settlement often means residential stability at the relocation destination. In this study, we used the self-reported frequency of visiting places of origin to measure settlement. In the post-relocation survey in 2019, we asked “How often did you visit the places of origin after your relocation?” These answers were coded from 1 to 3, where 1 represents “never,” 2 represents “sometimes,” and 3 represents “often.” Answers to this question were used to represent settlement status. An important note is that while we used “visit” and “return” interchangeably, neither a visit nor a temporary return to a place of origin is return migration, and it should not be interpreted as such in this context.

#### Independent variables

Our independent variables are household socioeconomic and demographic factors and community characteristics. The measurements of some of the independent variables such as age, gender, marital status, education, and household size are straightforward. Others are more conceptual and/or constructed through combinations of a set of variables that need additional explanation.

*Household income*. We calculated household income by subtracting household expenditures from cash income and market values of non-cash assets (e.g., agricultural and forestry products waiting to be sold).

*Apartment satisfaction*. We constructed apartment satisfaction by adding up 12 5-point scale indicators representing relocatees’ attitudes toward their destination government-built apartments and basic facilities such as apartment location and quality, water, electricity, road infrastructure, and recreational facilities. A larger number indicates more satisfaction with the apartment at the destination.

*Social networks*. Social networks, coupled with the social capital inherent in those social relationships, can manifest through shared values, trust, and cooperation [54]. Social networks have been shown to have an impact on population migration [55]. In a recent study investigating the association between farm size and social network formation among rice producers in China, Simpson [56] utilized the concept of friendship as a measure of social networks. We adopted a similar approach and defined close friends as individuals to whom participants expressed a willingness to lend more than 5,000 RMB (equivalent to approximately 724 USD at the 2016 and 2019 exchange rates), which is considered a threshold indicative of close relationships within the Chinese cultural context. The count of close friends was subsequently employed as a proxy to represent the social networks of the targeted relocatees. It is important to acknowledge that while this approach respects the cultural dimension of social networks in the Chinese context, it may not comprehensively capture the entirety of social networks as a broader conceptual construct.

### Modeling

We used the 2016 survey data to answer the research question concerning relocation willingness and combined the 2017 and 2019 survey data to explore the driving forces of settlement. Accordingly, we employed different modeling approaches for the relocation willingness model and the settlement model.

In the relocation model, we treated the dependent variables as ordinal variables and applied the multivariate ordinal logistic model (OLM) to the data we collected in 2016. The ordinal logistic regression model can be expressed as follows [57–59]:

$$logit\left(P\left(Y\leq j|X=x\right)\right)=ln\left(\frac{P(Y\leq j|X=x)}{1-P(Y\leq j|X=x)}\right)=\alpha_{j}+(-\beta_{1}x_{1}-\beta_{2}x_{2}-\ldots -\beta_{k}x_{k})$$ (1)

where *P*(*Y* ≤ *j*│*X* = *x*) is the cumulative probability that the dependent variable *Y* is in category *j* or a category less than *j*; *α*_*j*_ is the threshold for category *j*; *β*_1_, *β*_2_, …, *β*_*k*_ are the coefficients for the independent variables *x*_1_, *x*_2_, …, *x*_*k*_; and *ln* is the natural logarithm.

As previously mentioned, the survey data were collected hierarchically, with households nested within counties, and counties nested within provinces. To address the hierarchical structure and clustering effect in the data, we employed a three-level (household-, county-, and province-level) multilevel model (MLM) for the relocation model. The MLM follows the general form as follows [60]:

$$Y_{ijk}=\gamma_{000}+\gamma_{100}X_{ijk}+\mu_{0jk}+\mu_{00k}+\epsilon_{ijk}$$ (2)

where *Y*_*ijk*_ is the settlement for the *ith* household in the *jth* county within the *kth* province; *γ*_000_ is the overall intercept, representing the expected value of *Y* when all predictors are at 0; *X*_*ijk*_ represents a vector of covariates; *μ*_0*jk*_ and *μ*_00*k*_ are the random intercept for level 2 (county) and level 3 (province), capturing the deviation of each level 2 and level 3 unit’s average outcome from the overall average, respectively; and *ϵ*_*ijk*_ is the level 1 (household) residual term, representing the deviation of household observations from their expected value based on the level 2 and level 3 group averages. It should be noted that in the relocation model, the covariates in Eqs 1 and 2 include household head’s characteristics and household’s characteristics such as its economic conditions, characteristics related to infrastructure accessibility, social network at the origin community, and household’s familiarity with the PARP project.

For the settlement model, we employed a similar approach as in the relocation model, using OLM and MLM to fit the data. Moreover, as these modeling techniques do not explicitly consider the panel features of the settlement model, which comprises two waves of surveys, we combined the 2017 and 2019 survey data, creating a panel dataset. Subsequently, we applied fixed-effects (FE) models to explore the driving forces behind the settlement patterns of relocatees. The panel data fixed-effects model includes all the explanatory variables in the earlier analyses that are time-varying between the survey years 2017 and 2019, including household income, household head age, household size, left-behind family members, friends at the destination and origin, and apartment satisfaction. For the explanatory variables in the earlier analyses that are time-invariant (such as household head’s gender, education, marital status, and ethnicity, and certain household characteristics such as livestock values, landholding size, relocation preference, and commuting time between the origin and destination communities), we created interaction terms between these variables with a dummy variable for the 2019 survey year and included the interaction terms in the settlement model. Including those interaction terms provides two advantages: it allows time-invariant variables to be included in the analysis, as fixed-effects models typically drop them; and more importantly, it enables us to estimate how the impact of these time-invariant variables on settlement changed in 2019 relative to in 2017. For instance, a household with a married couple could be more likely to settle at the destination the longer they have been relocated. The panel models follow the general form as follows:

$$Y_{it}=\alpha +\beta X_{it}+\mu_{i}+v_{t}+\varepsilon_{it}$$ (3)

where *Y*_*it*_ represents settlement for household *i* at time *t*, *α* is the intercept, *X*_*it*_ is a vector of covariates, *μ*_*i*_ is the household-fixed effect, *v*_*t*_ is the time-fixed effect, and *ε*_*it*_ is the error term. We also employed FE models with county- and province-clustered standard errors, accounting for potential correlation in error terms at the county and province levels, respectively. As mentioned above, the covariates in the settlement model in Eq 3 include those time-varying explanatory variables and the interaction terms of those time-invariant explanatory variables and a dummy variable for the survey year 2019.

As stated in the hypotheses, our particular interest in the relocation model is to examine the effects of basic infrastructure accessibility and landholding size on relocation willingness. These two factors play important roles in influencing the households’ livelihoods, well-being, and relocation decision-making processes, and can therefore significantly impact relocation decisions. In the settlement model, we focused on assessing the influence of relocation completeness on the settlement of relocated households at the destination. Relocation completeness refers to whether the household completely moved all its members. Specifically, we measured relocation completeness using the presence of left-behind household members at the origin. The completeness of relocation determines the integrity of the household, therefore influencing resettlement at the destination. Household income and livestock values were log-transformed before being included in the models. This transformation was necessary due to the large standard variations and the potential presence of outliers in the original measures. Since China is regionally diverse [61], we included county dummy variables both in the relocation preference and settlement models to control for hard-to-measure characteristics such as cultural differences and other unobserved heterogeneities. Note that we used relocation preference differently in the two models. In the relocation preference model, relocation preference was treated as the outcome variable, while in the settlement model, we treated relocation preference as a control variable to reveal the net effects of the key factors in question. The objectives were to explore factors that influence relocation preference and settlement in the subsidized relocation program, not to examine the effect of the government subsidy itself on the relocation process. The analyses were conducted using Stata 16.1.

## Results

### Descriptive statistics

In 2016 a significant proportion of households (75.68%) showed a high likelihood of relocating, while less than 1% expressed a disinclination to voluntarily leave their place of origin (Table 1). The sampled households were predominantly headed by males (93.01%) in their 50s, with a majority being married (78.80%) and having attained less than a middle school education (70.60%). The largest ethnic group in China—the Han people—made up the majority of household heads (69.90%). On average, households comprise approximately four individuals. The log-transformed average household income and livestock values were 6.33 and 1.52, with the original values before transformation being 1,313.24 and 257.33, respectively. The average landholding size was 0.31 hectares. In terms of basic infrastructure, most households had access to electricity (94.36%), while a smaller proportion had access to running water (52.84%). The average distance to the nearest paved road and local market was 2.18 km and 10.78 km, respectively. As for educational facilities, the average distance was 7.15 km to the nearest elementary school, 16.00 km to the nearest middle school, and 54.25 km to the nearest high school. On average, households reported having 27.77 friends and had been visited approximately 4.47 times by government officials regarding the relocation policy at the time of the 2016 survey.

**Table 1:** Descriptive statistics for variables used in the relocation preference and settlement models in the Poverty Alleviation Relocation Program (PARP), 2016–2019.

	2016		2017		2019	
	Mean/%	SD	Mean/%	SD	Mean/%	SD
Relocation preference						
Not likely	0.61%	—	—	—	—	—
Less likely	4.43%	—	—	—	—	—
Undecided	2.66%	—	—	—	—	—
Somewhat likely	16.64%	—	—	—	—	—
Very likely	75.68%	—	—	—	—	—
Visiting frequency^a^						
Never	—	—	21.68%	—	33.83%	—
Sometimes	—	—	35.16%	—	30.17%	—
Always	—	—	43.16%	—	36.01%	—
Household head age	52.95	12.50	53.21	12.09	54.40	12.22
Household head gender						
Male	93.01%	—	92.21%	—	93.03%	—
Female	6.99%	—	7.79%	—	6.97%	—
Household head marital status						
Single	21.20%	—	19.37%	—	22.06%	—
Married	78.80%	—	80.63%	—	77.94%	—
Household head education, middle school and above						
No	70.60%	—	75.37%	—	69.49%	—
Yes	29.40%	—	24.63%	—	30.51%	—
Household head, Han people						
No	30.10%	—	22.74%	—	28.68%	—
Yes	69.90%	—	77.26%	—	71.32%	—
Household size	3.81	1.52	4.11	1.71	4.28	1.61
Household income (log)	6.33	2.04	7.31	1.27	6.28	1.39
Livestock value at the origin (log)	1.52	2.83	1.87	3.14	1.34	2.87
Landholding size at the origin	0.31	0.39	0.48	0.77	0.58	1.55
Number of friends at the origin	27.77	40.45	26.05	31.27	26.39	32.72
Running water at the origin						
No	47.16%	—	—	—	—	—
Yes	52.84%	—	—	—	—	—
Electricity at the origin						
No	5.64%	—	—	—	—	—
Yes	94.36%	—	—	—	—	—
Distance to the nearest paved road	2.18	3.05	—	—	—	—
Distance to the nearest local market	10.78	7.58	—	—	—	—
Distance to the nearest elementary school	7.15	7.35	—	—	—	—
Distance to the nearest middle school	16.00	12.60	—	—	—	—
Distance to the nearest high school	54.25	38.18	—	—	—	—
Number of times visited by officials	4.47	4.32	—	—	—	—
Landholding size at the destination	—	—	0.01	0.05	0.04	0.15
Apartment satisfaction at the destination	—	—	51.49	8.17	48.05	7.20
Left-behind members						
No	—	—	90.53%	—	94.16%	—
Yes	—	—	9.47%	—	5.84%	—
Number of friends at the destination	—	—	13.74	20.25	24.79	23.71
One-way commuting time in minutes	—	—	48.47	66.22	55.94	62.88
N	2,146		475		1,147	

*Note*. For categorical variables, we reported the percentage of each category. Household income and livestock values were converted from RMB to USD using the yearly average exchange rates provided by the IRS, where the average exchange rate was 1:6.91 in 2016. Landholding size was converted from mu, a Chinese measure of land area, to hectare (ha), where 1 mu equals approximately 0.07 ha. A negligible proportion of the households (only 3%) experienced changes in their household head between 2017 and 2019, presumably because of self-reporting errors or outliers.

^a^ The reasons for visiting places of origin are farming (57.73%), socializing (26.28%), herding (7.66%), residing (1.59%), and other reasons (6.74%).

Regarding settlement patterns, a substantial proportion (78.32% in 2017 and 66.18% in 2019) of relocated households reported always or sometimes visiting their place of origin after the relocation. The primary reasons for their visits were farming (57.73%), socializing (26.28%), herding (7.66%), and other reasons (6.74%), with a minimal percentage (1.59%) using former residence in their place of origin as a temporary residence while visiting. The characteristics of the households, their economic situations, and their social networks remained similar for relocated households in 2017 and 2019 compared with their status prior to relocation in 2016. The only notable difference was observed in landholding size. At places of origin, the average landholding size was 0.48 hectares in 2017 and 0.58 hectares in 2019, while at relocation destinations, the average landholding size was 0.01 hectares in 2017 and 0.04 hectares in 2019. Among the relocated households, 9.47% in 2017 and 5.84% in 2019 had left-behind household members at their place of origin. The average satisfaction with apartments in the destination community was 51.49 in 2017, which decreased to 48.05 in 2019. Typically, the relocation destinations were within walking distance of approximately 48.47 and 55.94 minutes from their places of origin in 2017 and 2019, respectively.

**Fig 3:**
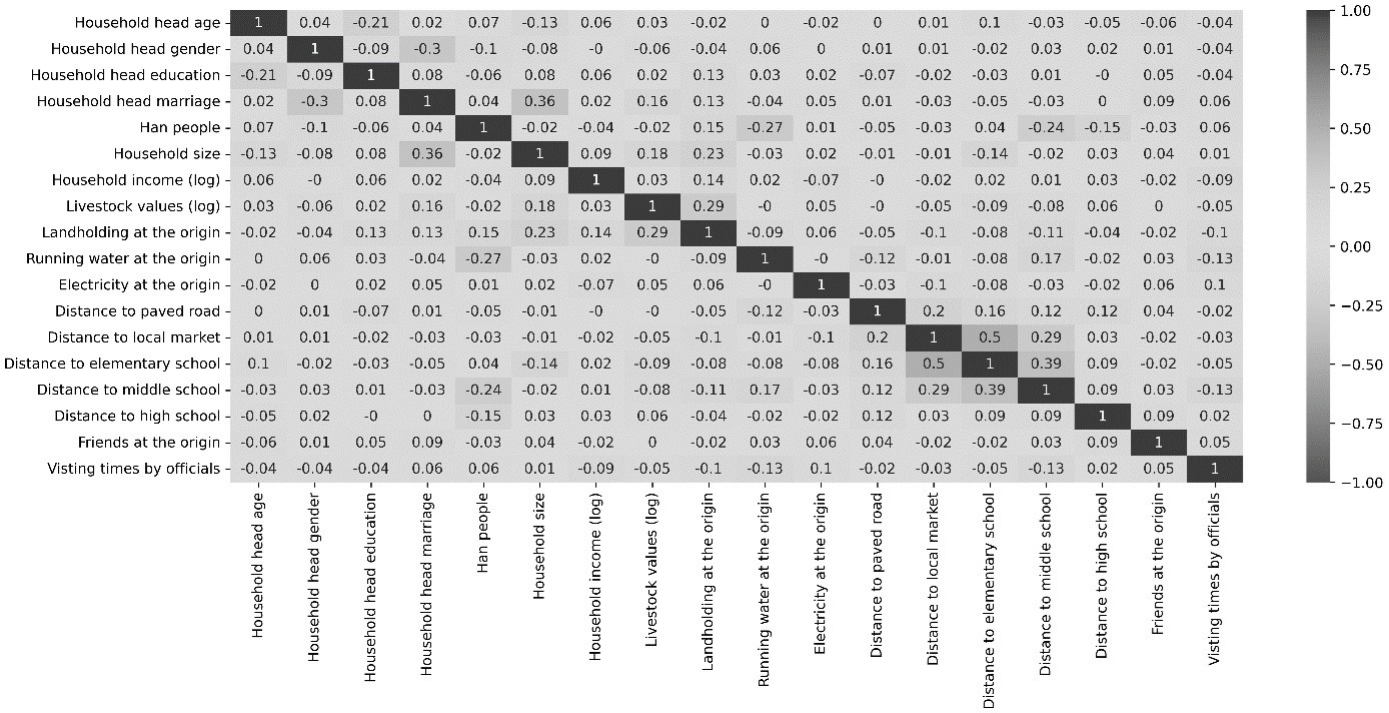
Correlation matrix of the covariates in the relocation preference model in the Poverty Alleviation Relocation Program (PARP) in China, 2016.

**Fig 4:**
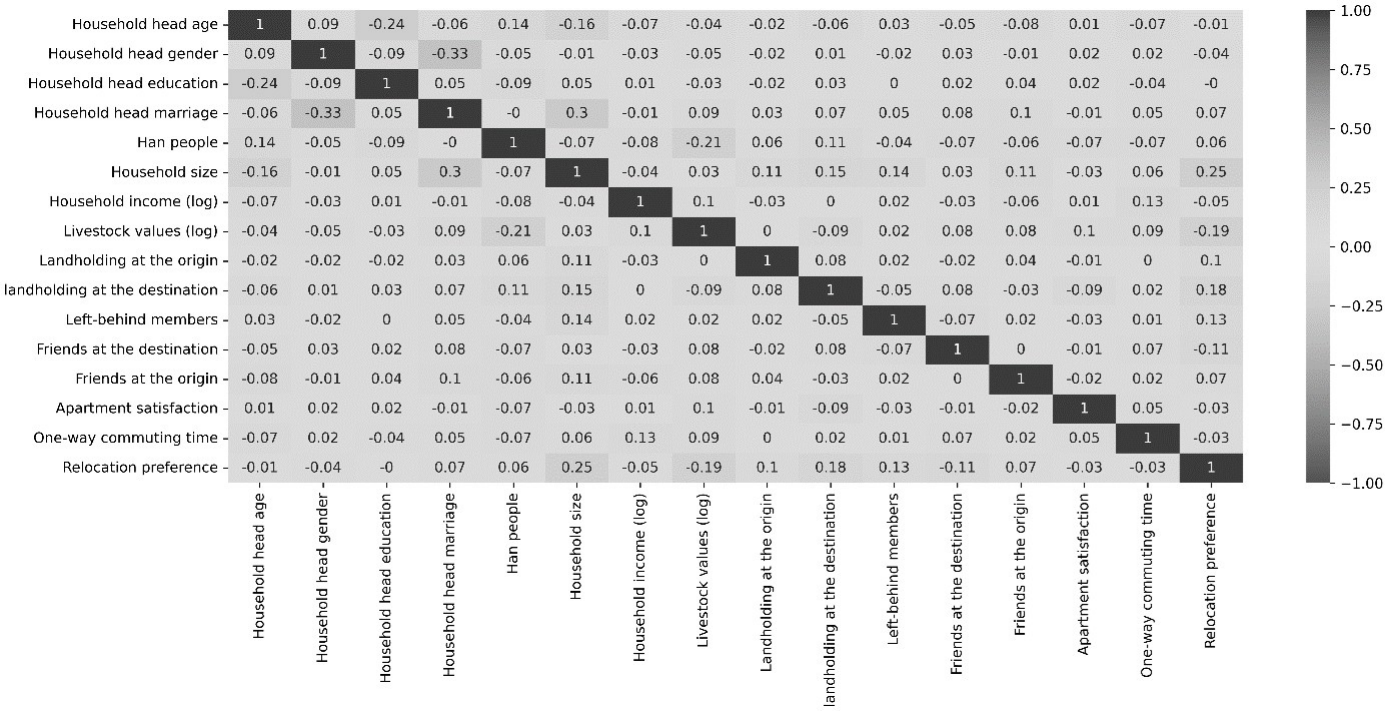
Correlation matrix of the covariates in the settlement model in the Poverty Alleviation Relocation Program (PARP) in China, 2017–2019.

Figs 3 and 4 present the correlation matrices of the covariates in the relocation preference and settlement models. Except for the correlation between the distance to the local market and elementary school, which is 0.5, all other correlations show small magnitudes. These findings indicate that there is no significant multicollinearity issue among the covariates in the models.

### Relocation preference model

We employed the stepwise estimation approach by adding household head demographic, household socioeconomic factors, and community characteristics in the models at each step using OLM and three-level MLM (Table 2).

**Table 2:** Relocation preference model in the Poverty Alleviation Relocation Program (PARP) in 2016.

	OLM			MLM		
	Model 1	Model 2	Model 3	Model 4	Model 5	Model 6
Household head age	-0.003	-0.003	-0.003	-0.003	-0.003	-0.003
Household head gender, female	0.222	0.222	0.245	0.220	0.222	0.247
Household head education, middle school, yes	-0.033	-0.020	0.006	-0.043	-0.029	-0.009
Household head marital status, married	0.240	0.277	0.288*	0.242	0.283*	0.294*
Household head Han people, yes	-0.357	-0.364	-0.309	-0.369	-0.371	-0.310
Household size		-0.006	-0.002		-0.009	-0.005
Household income (log)		-0.009	-0.012		-0.010	-0.013
Livestock values at origin (log)		-0.008	-0.011		-0.006	-0.008
Landholding size at origin		-0.301	-0.296		-0.301	-0.288
Running water at origin, yes			0.066			0.066
Electricity at origin, yes			-0.317			-0.313
Distance to paved road at origin			0.086**			0.090**
Distance to market at origin			-0.005			-0.005
Distance to elementary school at origin			0.022*			0.022*
Distance to middle school at origin			-0.006			-0.007
Distance to high school at origin			2e-4			0.001
Friends at origin			-0.001			-0.001
Number of times visited by officials			0.018			0.020
County dummy	Yes	Yes	Yes	—	—	—
Observations	2,146	2,146	2,146	2,146	2,146	2,146
Pseudo R-squared	0.109	0.110	0.117	—	—	—
AIC	3,010	3,014	3,011	3,047	3,051	3,046
BIC	3,146	3,173	3,221	3,110	3,136	3,183
ICC of province	—	—	—	0.185	0.175	0.159
ICC of county	—	—	—	0.291	0.286	0.256

*Note*.

*** p < 0.001,

** p < 0.01,

* p < 0.05 (two-tailed test).

AIC = Akaike’s information criterion; BIC = Schwartz’s Bayesian information criterion; ICC = intraclass correlation coefficient.

Models 1 and 2 include only the household head demographics and household characteristics, respectively. We found that household head demographics and household characteristics were not significant predictors of relocation preference at the household level. Model 3 is the full model with all covariates. The results showed that marital status and basic infrastructure were positively associated with relocation preference. On the one hand, married couples are more likely to relocate, a finding that may be explained by life course considerations that suggest married couples are more likely to seek out places with promising economic prospects [62, 63]. On the other hand, basic infrastructure, particularly longer distances to paved roads and elementary schools at the place of origin, increased the probability of relocation. This echoes the classic push—pull factors that influence migration decision-making, as adverse living conditions in a place of origin can trigger out-migration. Landholding size decreased relocation preference, however, such effects were insignificant.

Models 4 through 6 present the results from the multilevel model, which are generally consistent with the findings from the full model using OLM. This indicates the robustness of our findings. In sum, our results provide evidence supporting Hypothesis 1—that basic infrastructure increases the probability of relocation. Nevertheless, no strong evidence supporting Hypothesis 2—that landholding size at places of origin will negatively affect relocation preference.

### Settlement model

We first employed OLM and MLM to analyze the settlement patterns of relocatees after their relocation. The OLM considers the ordered nature of the dependent variable, and the adoption of MLM was motivated by the need to address clustering effects arising from the hierarchical structure of the dataset (Table 3).

**Table 3:** Settlement model in the Poverty Alleviation Relocation Program (PARP), 2017–2019.

	OLM			MLM		
	Model 7	Model 8	Model 9	Model 10	Model 11	Model 12
Household head age	0.005	0.002	0.002	0.004	0.001	0.002
Household head gender, female	-0.143	-0.051	-0.063	-0.118	-0.018	-0.061
Household head education, middle school, yes	0.117	0.103	0.079	0.111	0.105	0.086
Household head marital status, married	0.374**	0.318*	0.327*	0.373**	0.320*	0.306*
Household head Han people, yes	0.551**	0.544**	0.542**	0.366*	0.359*	0.348*
Household size		0.032	0.035		0.031	0.041
Household income (log)		0.012	0.013		0.049	0.017
Livestock values at origin (log)		0.071***	0.072***		0.072***	0.070***
Landholding size at origin		0.079	0.076		0.062	0.067
Landholding size at destination		-0.858	-0.900		-1.109*	-1.000*
Left-behind members, yes		1.415***	1.432***		1.481***	1.476***
Friends at destination		-0.003	-0.003		-0.004*	-0.003
Friends at origin		-1e-4	-1e-4		-2e-4	-7e-5
Relocation preference, less likely			0.718			0.739
Relocation preference, undecided			1.355			1.425
Relocation preference, somewhat likely			0.786			0.839
Relocation preference, very likely			0.944			0.967
Apartment satisfaction at destination			-0.006			-0.006
One-way commuting time			-0.002			-0.002
County dummy	Yes	Yes	Yes	—	—	—
Year dummy	Yes	Yes	Yes	Yes	Yes	Yes
Observations	1,622	1,622	1,622	1,622	1,622	1,622
Pseudo R-squared	0.043	0.064	0.066	—	—	—
AIC	3,440	3,383	3,386	3,481	3,409	3,407
BIC	3,564	3,550	3,586	3,524	3,495	3,531
ICC of province	—	—	—	5.15e-37	2.75e-34	9.80e-34
ICC of county	—	—	—	0.088	0.063	0.063

*Note*.

*** p < 0.001,

** p < 0.01,

* p < 0.05 (two-tailed test).

AIC = Akaike’s information criterion; BIC = Schwartz’s Bayesian information criterion; ICC = intraclass correlation coefficient.

Regardless of the modeling approaches and specifications, we consistently found that households with left-behind members were more likely to revisit their place of origin compared with households without left-behind members. Furthermore, the results also revealed that higher livestock values at the place of origin were associated with an increased frequency of relocatees’ visits. This evidence supports Hypothesis 3—that the completeness of the relocation process is an important factor affecting the settlement of relocatees at their destination.

To leverage the panel data structure while acknowledging potential correlation within geographical units, we then estimated panel data FE models with county- and province-clustered standard errors (Table 4). Consistent with results in Table 3, left-behind members play a crucial role in settlement (Models 10 through 12), with households with left-behind members being more likely to temporarily visit their places of origin after relocation. However, another variable of interest, livestock values, became less important in 2019 than in 2017, as reflected in the insignificant results of the variable. Collectively, these findings further support Hypothesis 3, that the presence of left-behind members was a primary concern for relocated households, significantly influencing their settlement and prompting relocatees to temporarily return to care for the left-behind members. The consistent results regarding the directions and significances of the interaction terms suggested robustness, irrespective of the various clustering options employed in the FE models. In addition, we found that the coefficient of the interaction term between household head’s marital status and survey year 2019 is significantly positive, indicating that a household with a married couple is more likely to settle at destination in 2019 than in 2017.

**Table 4:** Settlement model using panel data fixed-effects regression with different clustering options in the Poverty Alleviation Relocation Program (PARP), 2017–2019.

	Model 10	Model 11	Model 12
	No clustering	County-clustered SE	Province-clustered SE
Household income (log)	0.019	0.019	0.019
	(0.081)	(0.058)	(0.071)
Household head age	-0.021	-0.021	-0.021
	(0.029)	(0.031)	(0.030)
Household size	0.230	0.230	0.230
	(0.195)	(0.143)	(0.125)
Left-behind members, yes	1.584**	1.584*	1.584*
	(0.598)	(0.723)	(0.670)
Friends at destination	-0.002	-0.002	-0.002
	(0.005)	(0.005)	(0.006)
Friends at origin	-0.155***	-0.155***	-0.155***
	(0.014)	(0.017)	(0.015)
Apartment satisfaction at destination	0.005	0.005	0.005
	(0.018)	(0.019)	(0.014)
Household head gender × survey year 2019	1.045	1.045*	1.045***
	(0.646)	(0.449)	(0.086)
Household head education × survey year 2019	-0.075	-0.075	-0.075
	(0.360)	(0.466)	(0.433)
Household head marital status × survey year 2019	1.135*	1.135*	1.135*
	(0.518)	(0.546)	(0.501)
Household head Han people × survey year 2019	-0.789	-0.789	-0.789**
	(0.412)	(0.524)	(0.291)
Livestock values at origin (log) × survey year 2019	0.097	0.097	0.097
	(0.053)	(0.051)	(0.072)
Landholding size at origin × survey year 2019	0.074	0.074	0.074
	(0.419)	(0.252)	(0.309)
Landholding size at destination × survey year 2019	-0.463	-0.463	-0.463
	(1.256)	(1.457)	(1.240)
Relocation preference, not likely × survey year 2019	-14.595***	-14.595***	-14.595***
	(1.272)	(1.609)	(1.048)
Relocation preference, less likely × survey year 2019	-2.791*	-2.791*	-2.791*
	(1.329)	(1.371)	(1.384)
Relocation preference, undecided × survey year 2019	-14.214***	-14.214***	-14.214***
	(1.511)	(1.364)	(1.475)
Relocation preference, somewhat likely × survey year 2019	-1.388	-1.388	-1.388*
	(0.850)	(0.731)	(0.545)
Relocation preference, very likely × survey year 2019	-1.298	-1.298	-1.298**
	(0.666)	(0.715)	(0.469)
One-way commuting time × survey year 2019	0.005	0.005***	0.005**
	(0.003)	(0.001)	(0.002)
Household dummy	Yes	Yes	Yes
County dummy	Omitted	Omitted	Omitted
Year dummy	Yes	Yes	Yes
Observations	604	604	604
Pseudo R-squared	0.190	0.190	0.190
AIC	379	379	379
BIC	467	467	467

*Note*.

*** p < 0.001,

** p < 0.01,

* p < 0.05 (two-tailed test).

SE = standard error. AIC = Akaike’s information criterion; BIC = Schwartz’s Bayesian information criterion.

## Discussion and conclusions

Using data collected before and during the PARP in China, currently the largest contemporary government-led relocation project worldwide, this study explored factors associated with people’s attitudes and preferences about subsidized relocation and settlement at the relocation destinations. The results revealed that while the majority of relocatees demonstrated a high level of relocation willingness, there was variation in the levels of relocation preference and settlement among them. These differences were influenced by community-level characteristics such as distance to basic infrastructure, as well as household-level factors such as relocation completeness status. Overall, we provided empirical evidence supporting the notion that inadequate basic infrastructure serves as a push factor for individuals to leave their original locations within the PARP, while incomplete relocation and the resulting disrupted family ties act as a pull factor, leading to a return to their place of origin and undermining the process of settlement within the destination community.

Our results support previous theoretical and empirical understandings of relocation while revealing context-specific dynamics of relocation and settlement within the PARP. The high level of relocation preference observed in the PARP aligns well with previous research demonstrating the positive influence of financial incentives on relocation participation [19]. Our findings also highlight the significant impact of community-level factors, particularly the distance to the nearest paved road, on relocation preference. This finding aligns with the classic push—pull theory and is consistent with previous empirical evidence that limited transportation accessibility encourages outward migration [64].

Contrary to Hypothesis 2, we did not find strong evidence supporting the hypothesized relationship between relocation preference and landholding size in the sending communities. This finding suggests that in a rapidly developing world with increasing income sources and diverse livelihood strategies such as off-farm employment [65], farmland does not necessarily serve as a strong anchor tying individuals to their place of origin. Moreover, our findings indicate that the completeness of the relocation process significantly influences settlement outcomes. This underscores the significance of strong family ties in Chinese society, emphasizing the integral nature of familial relationships [66]. It further highlights that the stability of settlement may be compromised when these ties are disrupted, as observed in the PARP, where household members were separated between their places of origin and relocation destinations.

Our analyses have limitations. First, relocation disrupts the sense of attachment, familiarity, and identity of the relocatees, potentially decreasing the study participation rate among certain demographic groups; previous studies have shown that elderly people and households with children are less likely to relocate [27, 67]. However, the PARP witnessed a high rate of relocation preference (75.68% of the sampled households in 2016 reported they were very likely to relocate), likely because of the universally available government subsidies and the strong desire to move out of poverty-stricken and environmentally vulnerable regions. This can possibly bias the estimates of the relocation preference model upward.

An additional factor that may have influenced the results and conclusions is the absence of control for relocation distance. Previous research has established that relocation distance plays a crucial role in shaping relocation decision-making [33, 45]. The reason for this omission is that, during the initial stages of the relocation project, a considerable portion of the targeted population was unaware of their future relocation destination. Second, our analyses did not consider any selection bias that can potentially incur endogeneity, especially when considering the settlement for relocated households. During the three-year period of this study, 60% of the sampled households moved to the new settlements. There might be some characteristics—for example, their abilities and aspirations to move—that selected them into the relocation process and affected their settlement. Without considering selection bias and its effects, the interpretations of the results are confined to associations rather than causal relationships.

Third, the measure of the social network using the number of close friends may be regarded as a simplified representation, considering that the concept of social network encompasses multiple dimensions and complexities. Social networks involve not only the number of close friends but also the structure, composition, and quality of relationships, as well as the various types of social ties within an individual’s social sphere. Therefore, capturing the full richness of social network dynamics requires a more comprehensive assessment that accounts for the diverse dimensions and nuances inherent in social interactions and connections.

Our findings have profound implications for government-led relocation projects and future studies. First, relocation is by no means the end of the story but the beginning [30]. What should follow are studies to find ways to promote relocatees’ social integration into new neighborhoods, skill-building for surviving and thriving, and assessing the long-term impacts on households and their offspring’s poverty status, health, and educational achievement. These research agendas are as important as the study of relocation itself.

Second, the study of government-led relocation calls for interdisciplinary efforts among geography, demography, and sociology. For instance, geographers can contribute to the selection of suitable relocation destinations and facilitate the relocation processes by disentangling the effects of “the sense of place” in forming identity and connecting people with landscapes [68], as well as the political and ecological factors that influence governance and decision-making [69]. Demographers and sociologists can develop and assess ways to promote social integration and socioeconomic well-being after relocations.

Last but not least, it is important to acknowledge the significant influence of environmental change and variability on global migration patterns. These factors have played a crucial role in driving migration worldwide and are projected to lead to displacement of an estimated 200 million people by the year 2050 [70]. Taking this into account, more work should be done to investigate the impacts of environmental change and variability on livelihood strategies and migration patterns in regions affected by different types of environmental factors.

## Supporting information

S1 File Poverty Alleviation Relocation Program (PARP) survey instrument (excerpt). (PDF)
