# Supplementary material for: Relocation preference and settlement: Lessons from the Poverty Alleviation Relocation Program in China
Source: PLoS One. 2024 Oct 4;19(10):e0309534. doi: 10.1371/journal.pone.0309534 (PMC11452026; doi:10.1371/journal.pone.0309534)
Supplement: S1 File — (PDF) [file pone.0309534.s001.pdf]

S1 File

Poverty Alleviation Relocation Program (PARP) survey instrument (excerpt).

Household ID

**Province name**		**Province code**	
**County name**		**County code**	
**Township name**		**Township code**	
**Village name**		**Village code**	
**Household head name**		**Household ID**	
**Interviewer's name**		**Interviewer's code**	
**Interview start time**		**Interview end time**	
**Reviewer's name**		**Reviewer's code**	

A. Household characteristics

	**A01**	**A02**	**A03**	**A04**	**A05**	**A06**
	Person ID	Gender	Relationship to the household head	Marital status	Race/ethnicity	Educational achievement
1						
2						
3						
4						
5						
6						
7						
8						
9						
10						

B. Basic infrastructure

	Question	Options/Instructions	Answer
**B01**	Is there running water in your household?	0. No<br>1. Yes	
**B02**	Is there electricity in your household?	0. No<br>1. Yes	
**B03**	How frequent are power outages in your household?	1. No power outages<br>2. Sometimes outages<br>3. No electricity at all	
**B04**	How far is it from your house to the nearest paved road?	In kilometers	
**B05**	How far is it from your house to the nearest primary school?	In kilometers	
**B06**	How far is it from your house to the nearest middle school	In kilometers	
**B07**	How far is it from your house to the nearest high school	In kilometers	

C. Household economy and social capital

	Question	Options/Instructions	Answer
**C01**	How much farmland do you have in your household?	In mu	

**C02**	What are the values of your livestock?		
**C03**	How many people do you feel comfortable talking about personal affairs and would like to borrow from or lend money to (more than 5000 RMB) in your original community?		
**C04**	How many people in your household are currently attending college?		

D. Relocation

	Question	Options/Instructions	Answer
**D01**	How do you know about the relocation project?	1. Government officials<br>2. Other villagers<br>3. TV, newspaper, and social media<br>4. Other sources (please specify)	
**D02**	How much would you like to relocate to the new settlement?	1. Very unlikely<br>2. Somewhat unlikely<br>3. Neutral<br>4. Somewhat likely<br>5. Very likely	
**D03**	How many times did government officials come and introduce the relocation project?		
**D04**	How many people do you feel comfortable talking about personal affairs and would like to borrow from or lend money to (more than 5000 RMB) in your relocation destination community?		
**D05**	How long does it take for you to go from your place of origin to the place of relocation destination with the most commonly used way for transportation?	In minutes	
**D06**	How often did you visit the places of origin after your relocation?	1. Never<br>2. Sometimes<br>3. Often	
**D07**	What is the primary reason for your visiting the places of origin?	1. Farming<br>2. Herding<br>3. Socializing<br>4. Residing<br>5. Other (please specify)	
**D08**	On a scale of 1 to 5, where 1 means very unsatisfied and 5 means very satisfied, how do you rate the location of the apartment in your relocation destination community?		
**D09**	On a scale of 1 to 5, where 1 means very unsatisfied and 5 means very satisfied, how do you rate the quality of the apartment in your relocation destination community?		
**D10**	On a scale of 1 to 5, where 1 means very unsatisfied and 5 means very satisfied, how do you rate the basic infrastructure in your relocation destination community?		
